# Yes but no

**DOI:** 10.1016/j.inpm.2022.100110

**Published:** 2022-06-18

**Authors:** Josh Levin

**Affiliations:** Stanford University School of Medicine, USA

Yes but no. Coined by my nephew at the age of 4, this phrase refers to things that are and are not at the same time. Bupivacaine lasts longer than lidocaine. But as Schneider et al. demonstrated in this issue of *Interventional Pain Medicine* [1], it does not. Yes but no.

In the process of selecting patients for medial branch radiofrequency neurotomy, physicians perform - and perhaps more importantly interpret - diagnostic medial branch blocks. Such interpretation, while seemingly straight-forward, is most certainly not. Definitions of a positive response vary greatly among practitioners, with some advocating for a single diagnostic block with ≥50% relief in pain [2], and others recommending dual-diagnostic blocks with complete relief of pain [3]. Even triple, placebo-controlled blocks have been supported [4], yet in recognition of the logistical challenges associated with placebo-controlled procedures, the Spine Intervention Society has recommended dual comparative blocks alternating between short and long lasting local anesthetics [5]. By requiring longer-lasting relief from a longer-acting medication, the use of a placebo can be avoided, provided that the patient does not know which local anesthetic was used. All that is required is that the long-acting anesthetic lasts longer. No problem, right?

Medical reversal refers to the phenomenon of changing well-established practices due to new and contradictory evidence [6]. Treating hypertension with atenolol [7], stable coronary artery disease with stents [8], premature ventricular contractions (PVCs) with antiarrhythmic medications [9], and routine screening of woman aged 40–49 with mammography [10], are just a few examples of common practices that were reversed when new and better evidence emerged. Often times, medical reversal can have profound effects, as in the case of antiarrhythmics for PVCs, which were found to be associated with higher mortality rates [9]. Clearly, high quality science is necessary if we wish to advance our fields and provide the best care to our patients.

In the case of dual comparative medial branch blocks, one might ask why. Why would lidocaine last longer than bupivacaine? Given the pharmacokinetics, we expect it would not [11]. One might opine that local anesthetics have a more unpredictable duration of action in chronic pain states, as prolonged relief beyond the expected duration of conduction blockade often occurs [12]. But the answer to the question of why is that it is the wrong question. Instead of why, we should be asking what. What does the literature say? Schneider et al. provide an answer [1], and in the process, have contradicted years of conventional wisdom. As a result of their work, we must now question the utility of relying on concordant responses when interpreting dual diagnostic blocks.

So, does bupivacaine last longer than lidocaine? Any 4-year-old can explain it to you. Yes but no.

## Declaration of competing interest

The authors declare that they have no known competing financial interests or personal relationships that could have appeared to influence the work reported in this paper.
